# Influence of COVID-19 infection on early pregnancy outcomes in different periods around frozen embryo transfer

**DOI:** 10.1186/s12884-024-06646-1

**Published:** 2024-06-24

**Authors:** Yiling Ko, Luting Chen, Chengliang Zhou, Ji Xi, Yu Xiao, Xiaojun Chen

**Affiliations:** 1 Reproductive Medical Center, School of Medicine, The International Peace Maternity and Child Health Hospital, Shanghai Jiao Tong University, 910 Hengshan Road, Shanghai, 200030 China; 2 Shanghai Key Laboratory of Embryo Original Diseases, Shanghai, 200030 China; 3 Shanghai Jiao Tong University School of Medicine, Shanghai, 200025 China

**Keywords:** Assisted reproduction, Abortion, Corona virus disease 2019 (COVID-19), Frozen embryo transfer (FET), Pregnancy

## Abstract

**Purpose:**

The study aimed to investigate the potential influence of COVID-19 infection on embryo implantation and early development in women undergoing frozen embryo transfer (FET), with a specific focus on infections occurring at different periods around FET.

**Methods:**

A retrospective analysis was performed on women who had undergone FET during a period marked by a significant surge in COVID-19 infection in Shanghai. All enrolled women experienced their first documented COVID-19 infection around the time of FET, ensuring that infections did not occur prior to oocyte retrieval. Participants were categorized into six groups based on the timing of infection: uninfected, ≥ 60 days, < 60 days before FET, 0–14 days, 15–28 days, and 29–70 days after FET. Clinical outcomes were compared across these groups.

**Results:**

The infection rate among the total of 709 cases was 78.28%. Infected individuals exhibited either asymptomatic or mild symptoms. The ongoing pregnancy rates for the first four groups were 40.7%, 44.4%, 40.5%, and 34.2% (*P* = 0.709) respectively, biochemical pregnancy rates (59.1% vs. 61.1% vs. 67.6% vs. 55.7%, *P* = 0.471) and clinical pregnancy rates (49.6% vs. 55.6% vs. 55.4% vs. 48.1%, *P* = 0.749), all showed no significant differences. Early spontaneous abortion rates across all six groups were 18.3%, 20.0%, 25.0%, 28.9%, 5.4%, and 19.0% respectively, with no significant differences (*P* = 0.113). Multivariable logistic analysis revealed no significant correlation between the infection and ongoing pregnancy.

**Conclusion:**

Asymptomatic or mild COVID-19 infections occurring around FET do not appear to have a significant adverse impact on early pregnancy outcomes.

## Introduction

Novel coronavirus pneumonia, caused by COVID-19, emerged as a global infectious disease subsequent to the severe acute respiratory syndrome (SARS) outbreak. Primarily affecting the respiratory tract [1], the COVID-19 infection swiftly disseminated worldwide. Shanghai, a major city in China, implemented rigorous measures to curb virus transmission since the first confirmed case on January 20, 2020. In order to detect patients in time, the government organized free nucleic acid or antigen tests for people in the city almost every 3 days. By the latter half of 2022, with the waning virulence of the virus, the national epidemic prevention strategy transitioned towards achieving herd immunity. During this period, a substantial number of asymptomatic and mildly infected individuals emerged in Shanghai, estimated to encompass over half of the city's population, leading to the rapid attainment of herd immunity.

Studies have indicated that pregnant women infected with COVID-19 during the middle and late stages of pregnancy seldom exhibit severe symptoms, with minimal risk of vertical transmission [2]. Nonetheless, COVID-19 infection during these stages may adversely impact pregnancy, perinatal outcomes, and newborns, resulting in preterm delivery, pre-eclampsia, increased rates of caesarean sections, and heightened requirements for perinatal intensive care [2, 3]. Given the increased risk of preterm birth, pregnancy complications, and related factors, neonatal COVID-19 infection poses a significant threat to newborns' lives [2, 3].

The impacts of COVID-19 infection on pregnancy planning, maternal health, and perinatal outcomes undergoing assisted reproductive therapy (ART) are subjects of ongoing discussion. Existing research has explored the outcomes of women receiving embryo transfers during the global COVID-19 pandemic, revealing no significant differences in early pregnancy loss and ongoing pregnancy rates among groups (displaying symptoms suggestive of COVID-19 infection, those exposed to infected individuals, and those with positive COVID-19 tests) [4]. Throughout the pandemic, ART treatment outcomes for infertility patients appeared unaffected, apart from an initial increase in cancellation rates [5–8]. However, these studies, characterized by their observational nature, limited infection rates, small scale, and variable timing of COVID-19 infection concerning ART treatment, exhibit considerable heterogeneity and diminished credibility.

Given the challenge of swiftly eradicating infectious diseases and the prospect of their enduring coexistence, it is imperative to acknowledge that patients severely affected by COVID-19 cannot undergo immediate ART treatments. Yet, questions persist regarding the potential risks associated with asymptomatic or mild COVID-19 infection during the pandemic. Should embryo transfers be postponed? When is the optimal time for conception? Insufficient studies exist to address these queries comprehensively. The high infection rate witnessed in Shanghai from late 2022 to January 2023 offers a unique opportunity for this study. The research objective is to investigate the impact of asymptomatic or mild COVID-19 infection occurring at different periods before or after frozen embryo transfer (FET) on clinical outcomes, and to provide valuable recommendations for pregnancy assistance strategies during future infectious disease pandemics.

## Materials and methods

### Study design

This retrospective study focused on women undergoing FET at the Assisted Reproduction Center of the International Peace Maternity & Child Health Hospital (IPMCH) between October 1, 2022, and January 31, 2023. The subjects were followed up for up to 70 days after FET for information on COVID-19 infection and pregnancy outcomes. Ethical approval and consent were obtained from the Ethics Committee of IPMCH (No. GKLW-A-2023–029-01).

### Survey of COVID-19 infection

**Fig. 1:**
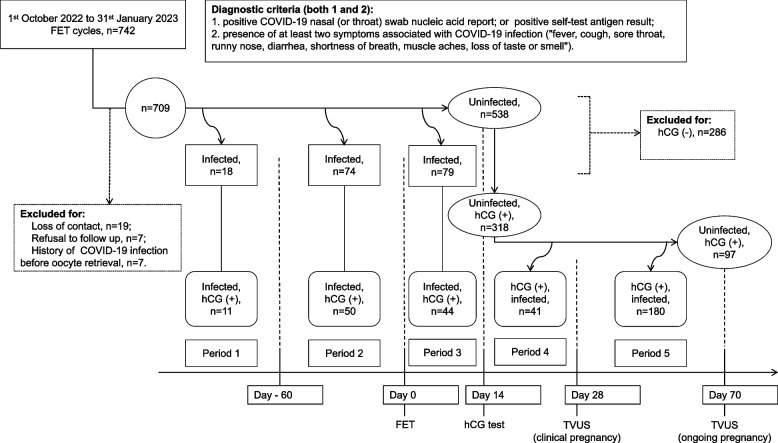
Flowchart. COVID-19, coronavirus disease 2019; FET, frozen embryo transfer; hCG, human chorionic gonadotropin; TVUS, transvaginal ultrasound

Information pertaining to COVID-19 infection, including infection date, symptoms, severity, detection method, and disease progression, was documented. In cases of clinical pregnancy after FET, the intensity and duration of morning sickness and instances of vaginal bleeding were recorded. Positive COVID-19 nasal (or throat) swab nucleic acid report, or positive self-test antigen result was necessary to confirm COVID-19 infection,when accompanied by at least two symptoms associated with COVID-19 infection including fever, cough, sore throat, runny nose, diarrhea, shortness of breath, muscle aches, loss of taste or smell, etc. Individuals who receive outpatient treatment for symptom relief and do not require hospitalization were diagnosed with a mild infection. [4, 9]. Refer to Fig. 1 for the study flowchart. Previous COVID-19 vaccination status of the women was also recorded.

As COVID-19 infections occur throughout the pre- and post-transfer periods, in order to study the impact of COVID-19 infections on biochemical, clinical, and ongoing pregnancy rates, participants were categorized into four groups based on the timing of infection: uninfected, ≥ 60 days before FET, < 60 days before FET, and 0–14 days after FET. Subsequently, in order to study the impact of COVID-19 infections on pregnancy loss within a pregnant cohort, the women were then divided into six groups:uninfected, ≥ 60 days before FET, < 60 days before FET, 0–14 days after FET, 15–28 days after FET, and 29–70 days after FET. It should be noted that the size of the uninfected group varied at different checkpoints, contingent upon the specific objectives of the analysis.

### Embryo assessment and cryopreservation

Oocytes were inseminated using conventional in vitro fertilization (IVF) or intracytoplasmic sperm injection (ICSI) methods. Embryo morphology was evaluated between Day 2 and Day 6 post-oocyte retrieval, and each embryo was graded according to the Istanbul consensus [10]. Embryos with good and fair grades were cryopreserved through vitrification.

### FET and luteal support

Various protocols were performed for FET cycles, including hormone replacement with or without down-regulation, ovarian stimulation, and natural cycles. Cleavage stage embryos were transferred on the second or third day after ovulation or progesterone administration if the endometrial thickness was ≥ 7mm. Blastocysts were transferred on the fifth or sixth day. The number (no more than two) of embryos transferred was determined based on clinician recommendations and patient preference. Luteal support was provided for 14 days after transfer, and then serum human chorionic gonadotropin (hCG) level was measured. Support continued for women with hCG levels > 10 IU/L, and clinical pregnancy was confirmed via transvaginal ultrasound (TVUS) performed 2–3 weeks later. Patients with clinical pregnancy were followed up for up to 70 days post-transfer, and luteal support extended for a minimum of 10 gestational weeks.

### Definition of outcomes

The primary outcome, ongoing pregnancy, was defined as an intrauterine live fetus beyond 12 weeks’ gestation. Secondary outcomes encompassed biochemical pregnancy (serum hCG level > 10 IU/L), clinical pregnancy (gestational sac presence on TVUS with rising hCG), loss of biochemical pregnancy (hCG reduction to normal before clinical pregnancy is established after biochemical pregnancy), and early spontaneous abortion (embryonic death or absence of fetal heartbeat post clinical pregnancy).

### Statistical analysis

SPSS software (IBM Corp.Armonk, NY, USA, V13.0) was used for data analysis. Numerical variables were presented as mean and standard deviation (SD), and categorical variables as number and percentage. ANOVA test compared groups for numerical variables, while Kruskal–Wallis test compared non-normally distributed data. Chi-squared test compared rates among groups. Multivariable logistic regression explored the correlation between COVID-19 infection periods and ongoing pregnancy. *P*-value less than 0.05 was considered significant.

## Results

Between October 1, 2022, and January 31, 2023, a total of 742 FET cycles were conducted at our center. Information on COVID-19 infection was collected for these women, with a loss rate of 4.45% (33 cases lost to follow-up). Of all the women followed, 400 had received 1–3 doses of the COVID-19 vaccine, while 309 had not. Since the Shanghai government had organized the city for free nucleic acid or antigen testing almost every 3 days from the onset of the outbreak until then, the timing of the onset of COVID-19 infection was clear. None of the women were infected with COVID-19 prior to oocyte retrieval. The subjects were followed for up to 70 days after FET. By the end of the study, there were 555 infections, resulting in a population infection rate of 78.28%. All infected individuals exhibited either asymptomatic or mild symptoms, and hospitalization due to COVID-19 infection was not required.

No significant differences were observed among the four groups containing uninfected, ≥ 60 days before FET, < 60 days before FET, and 0–14 days after FET when regarding baseline characteristics, including female age at the time of oocyte collection, infertility duration, body mass index (BMI), vaccination status, type of infertility, infertility diagnosis, ART method, endometrial preparation protocols, stage of transferred embryos, and number of transferred embryos (*P* > 0.05) (Table 1).

**Table 1:** General characteristics of participants

	Uninfected	Period of Coivd-19 infection^a^			*P*
		≥ 60 days before FET	< 60 days before FET	0—14 days after FET	
*N*	538	18	74	79	
Age (years)^b^	34.06 ± 4.41	32.83 ± 5.57	33.88 ± 4.21	34.75 ± 4.62	0.341
Infertility duration (years)	2.66 ± 2.35	2.75 ± 2.03	2.96 ± 2.26	2.69 ± 2.38	0.781
BMI (kg/m^2^)	23.32 ± 3.17	22.22 ± 3.28	21.65 ± 3.09	22.71 ± 3.23	0.217
Vaccination status (*n*, %)					0.162
Vaccinated	299 (55.6)	14 (77.8)	46 (62.2)	41 (51.9)	
Unvaccinated	239 (44.4)	4 (22.2)	28 (37.8)	38 (48.1)	
Type of infertility (*n*, %)					0.749
Primary	308 (57.5)	12 (66.7)	40 (54.1)	47 (60.3)	
Secondary	228 (42.5)	6 (33.3)	34 (45.9)	31 (39.7)	
Infertility diagnosis (*n*, %)					0.176
Tubal factor	178 (33.1)	5 (27.8)	30 (40.5)	24 (30.4)	
Ovulatory dysfunction	41 (7.6)	2 (11.1)	4 (5.4)	12 (15.2)	
Diminished ovarian reserve	41 (7.6)	1 (5.6)	5 (6.8)	10 (12.7)	
Endometriosis	28 (5.2)	1 (5.6)	6 (8.1)	3 (3.8)	
Male factor	82 (15.2)	7 (38.9)	8 (10.8)	7 (8.9)	
Both factors	118 (21.9)	2 (11.1)	13 (13.6)	18 (22.8)	
Unexplained	50 (9.3)	0 (0)	8 (10.8)	5 (6.3)	
ART method (*n*, %)					0.610
IVF	290 (53.9)	8 (44.4)	43 (58.1)	48 (61.5)	
ICSI	181 (33.6)	9 (50.0)	23 (31.1)	21 (26.9)	
PGT	67 (12.5)	1 (5.6)	8 (10.8)	9 (11.5)	
Endometrial preparation (*n*, %)					0.066
Natrual cycle	92 (17.1)	2 (11.1)	4 (5.4)	10 (12.7)	
Hormone replacement	120 (22.3)	6 (33.3)	20 (27.0)	27 (34.2)	
Hormone replacement with down-regulation	288 (53.5)	9 (50.0)	41 (55.4)	34 (43.0)	
Ovarian stimulation	38 (7.1)	1 (5.6)	9 (12.2)	8 (10.1)	
Stage of transferred embryos (*n*, %)					0.133
Cleavage	222 (41.3)	7 (38.9)	20 (27.0)	30 (38.0)	
Blastocyst	316 (58.7)	11 (61.1)	54 (73.0)	49 (62.0)	
Number of transferred embryos (*n*, %)					0.127
One	335 (62.3)	8 (44.4)	53 (71.6)	53 (67.1)	
Two	203 (37.7)	10 (55.6)	21 (28.4)	26 (32.9)	

*BMI* Body mass index, *FET* Frozen embryo transfer, *IVF* In vitro fertilization, *ICSI* Intracytoplasmic sperm injection, *N* Number, *PGT* Preimplantation genetic testing

^a^The participants' COVID-19 infection history was checked at the time point of Day 14 after embryo transfer

^b^Female age at the time of oocyte collection

### Analysis of COVID-19 impact on early pregnancy outcomes

To assess the impact of COVID-19 infection, pregnancy outcomes were compared across different groups. Biochemical pregnancy rates in the uninfected group, ≥ 60 days before FET, < 60 days before FET, and 0–14 days after FET were 59.1%, 61.1%, 67.6%, and 55.7%, respectively, with no significant differences (*P* = 0.471). Similarly, clinical pregnancy rates (49.6%, 55.6%, 55.4%, and 48.1%) and ongoing pregnancy rates (40.7%, 44.4%, 40.5%, and 34.2%) did not significantly vary among these groups (*P* = 0.749 for clinical pregnancy rates, *P* = 0.709 for ongoing pregnancy rates) (Table 2).

**Table 2:** Comparison of pregnancy outcomes between groups with different periods of Covid-19 infection

	Uninfected	Period of Covid-19 infection^a^			χ^2^	*P*
		≥ 60 days before FET	< 60 days before FET	0—14 days after FET		
*N*	538	18	74	79		
Biochemical pregnancy (*n*, %)	318 (59.1)	11 (61.1)	50 (67.6)	44 (55.7)	2.522	0.471
Clinical pregnancy (*n*, %)	267 (49.6)	10 (55.6)	41 (55.4)	38 (48.1)	1.218	0.749
Ongoing pregnancy (*n*, %)	219 (40.7)	8 (44.4)	30 (40.5)	27 (34.2)	1.383	0.709

*FET* Frozen embryo transfer, *N* Number

^a^The participants' COVID-19 infection history was checked at the time point of Day 14 after embryo transfer

### Analysis of COVID-19 impact on early pregnancy loss

Comparative analysis of biochemical pregnancy loss rates revealed no significant differences among the uninfected group, ≥ 60 days before FET, < 60 days before FET, 0–14 days after FET, and 15–28 days after FET (*P* = 0.678). Similarly, early spontaneous abortion rates did not significantly differ across the six groups (18.3%, 20.0%, 25.0%, 28.9%, 5.4%, and 19.0%, *P* = 0.113) (Table 3).

**Table 3:** Comparison of pregnancy loss rates between groups with different periods of Covid-19 infection

	Uninfected	Period of Covid-19 infection^a^					χ^2^	*P*
		≥ 60 days before FET	< 60 days before FET	0—14 days after FET	15—28 days after FET	29—70 days after FET		
Biochemical pregnancy loss (*n*, %)^b^	47/277 (17.0)	1/11 (9.1)	9/50 (18.0)	6/44 (13.6)	4/41 (9.8)	N/A	2.315	0.678
Early spontaneous abortion (*n*, %)^c^	15/82 (18.3)	2/10 (20.0)	10/40 (25.0)^d^	11/38 (28.9)	2/37 (5.4)	28/147 (19.0)^e^	8.914	0.113

*FET* Frozen embryo transfer, *N/A* Not applicable

^a^At the time point of Day 70 after embryo transfer, the COVID-19 infection history was checked for participants with positive hCG tests

^b^Only women with a positive hCG test were included in the analysis (*n* = 423)

^c^Only women in clinical pregnancy were included in the analysis (*n* = 356)

^d, e^One case of ectopic pregnancy was excluded from the statistical analysis

### Multivariable logistic regression analysis

After adjusting for potential influencing factors, no significant correlation was found between COVID-19 infection periods and ongoing pregnancy (*P* > 0.05). Factors significantly correlated with ongoing pregnancy included female age at the time of oocyte collection (OR = 0.948, 95% CI = 0.909–0.989, *P* = 0.014), blastocyst transfer (OR = 1.72, 95% CI = 1.174–2.520, *P* = 0.005), and the number of transferred embryos (OR = 1.955, 95% CI = 1.346–2.839, *P* < 0.001) (Table 4).

**Table 4:** Multivariable logistic regression of the correlation between Covid-19 infection periods and ongoing pregnancy

	B	OR	95% CI	*P*
Period of infection				
Uninfected	Contrast			
≥ 60 days before FET	0.056	1.058	0.365—3.068	0.918
< 60 days before FET	-0.002	0.998	0.539—1.851	0.996
0—14 days after FET	-0.355	0.701	0.366—1.341	0.283
15—28 days after FET	0.313	1.367	0.717—2.605	0.342
29—70 days after FET	-0.082	0.921	0.595—1.425	0.712
Age (years)^a^	-0.054	0.947	0.908—0.989	0.013
Stage of transferred embryos				
Cleavage	Contrast			
Blastocyst	0.540	1.716	1.172—2.515	0.006
Number of transferred embryos (*n*)				
One	Contrast			
Two	0.667	1.949	1.342—2.831	< 0.001

The analysis was adjusted for age, infertility duration, body mass index, vaccination status, infertility type, infertility diagnosis, ART method, endometrial preparation protocol, endometrial thickness, endometrial type, stage of transferred embryos and number of transferred embryos

*ART* Assisted reproductive technology, *FET* Frozen embryo transfer

^a^Female age at the time of oocyte collection

### Analysis of COVID-19 impact on early pregnancy adverse reactions

Among women achieving clinical pregnancy after FET (*n* = 355), there were no statistically significant differences in the incidence and severity of morning sickness or the occurrence of vaginal bleeding among the six groups (*P* > 0.05) (Table 5).

**Table 5:** Comparison of early pregnancy adverse reactions between groups with different periods of Covid-19 infection^a^

	Uninfected	Period of Covid-19 infection					χ^2^	*P*
		≥ 60 days before FET	< 60 days before FET	0—14 days after FET	15—28 days after FET	29—70 days after FET		
*N*	81	10	41	38	37	148		
Severity of morning sickness^b^ (*n*, %)							10.222	0.35
None	46 (56.8)	4 (40.0)	17 (41.5)	16 (42.1)	11 (29.7)	60 (40.5)		
Mild	33 (40.7)	6 (60.0)	23 (56.1)	21 (55.3)	24 (64.9)	83 (56.1)		
Moderate to severe	2 (2.5)	0 (0)	1 (2.4)	1 (2.6)	2 (5.4)	5 (3.4)		
Bleeding (*n*, %)	32 (39.5)	4 (40.0)	17 (41.5)	13 (34.2)	17 (45.9)	67 (45.3)	2.068	0.845

*FET* Frozen embryo transfer, *N* Number

^a^Only women in clinical pregnancy were included in the analysis

^b^The severity of morning sickness was determined based on the following criteria: (1) Individuals experiencing no nausea or only mild nausea without vomiting were categorized as having no symptoms of early pregnancy; (2) persistent vomiting (≥ 3 times per day), weight loss of ≥ 5% relative to pre-pregnancy weight, and a positive urine ketone body test were classified as moderate to severe (with complications referred to as severe), necessitating clinical intervention [11]; (3) those not meeting the above criteria and not requiring medicinal treatment were classified as having mild symptoms

## Discussion

Previous studies have explored the impact of COVID-19 infection on pregnancy outcomes, with some focusing on the recovery period post-infection and its effects on oocyte maturation, embryo quality and ART outcomes [8]. Our study aimed to investigate the potential effect of COVID-19 infection on embryo implantation and early development, and uniquely, concentrated on women who received FET and were not infected prior to oocyte retrieval, thereby excluding the effect of COVID-19 infection on oocytes. This study benefited from the clear timeline of both FET and COVID-19 infection, ensuring precise categorization of infected individuals. The infection rate in our study population was notably high (78.28%), allowing for a comprehensive analysis of the impact of COVID-19 infection at various periods.

In this study, no significant differences were found in embryo implantation and early development, including biochemical pregnancy rate, clinical pregnancy rate, and ongoing pregnancy rate, among the four groups (uninfected, ≥ 60 days before FET, < 60 days before FET, 0–14 days after FET). These findings suggest that even asymptomatic or mild COVID-19 infection, occurring during the critical period of embryo implantation (0–14 days after transplantation), does not significantly impact embryo implantation into the endometrium. Moreover, it implies that such infections do not affect early embryo development or maternal endometrial receptivity [12]. Multivariable logistic regression analysis reinforced these findings, revealing that ongoing pregnancy rate was significantly correlated with factors such as maternal age, stage of transferred embryos, and the number of transferred embryos, while COVID-19 infection did not exhibit a significant correlation. It is consistent with the outcomes of related studies [13, 14]. Additionally, our study indicated that asymptomatic or mild COVID-19 infection did not increase the occurrence of early pregnancy adverse reactions, such as severe morning sickness or vaginal bleeding, following the establishment of early clinical pregnancy.Comparison with prior studies highlights the unique aspects of our research.

A study from Israel with a smaller sample size (n = 82) assessed the impact of COVID-19 infection on pregnancy rates in FET. It reported a lower clinical pregnancy rate for women who underwent embryo transfer within 60 days post-infection compared to those who waited longer. Therefore, the authors recommended to delay embryo transfer for at least 60 days after COVID-19 recovery in FET cycles for women with limited embryo number [15]. However, our study, conducted during a concentrated outbreak period, featured a larger sample size and a higher population infection rate. Notably, the lowest clinical pregnancy rate in infected individuals in our study was 48.1%, substantially higher than the rates reported in previous studies. This discrepancy led us to speculate that asymptomatic or mild COVID-19 infection at various periods before and after FET does not significantly impair the pregnancy rate of FET. Consequently, delaying pregnancy plans for prior infection might not be necessary. However, these conclusions are preliminary and require cautious interpretation, urging further research for clarification.

During the global COVID-19 pandemic, various studies have explored the relationship between COVID-19 infection and pregnancy outcomes, presenting diverse findings. A prospective cohort study in the UK demonstrated an increased risk of early abortion in women infected with COVID-19 before 13 weeks’ gestation [16]. However, a retrospective cohort study in New York City did not find an elevated rate of pregnancy failure within the first trimester among women infected with COVID-19 who achieved pregnancy through ART [4]. Similarly, a single-center study in Iran found no negative impact on ART outcomes despite symptoms of infection occurring before or after the ART cycle [5]. In India, a retrospective analysis showed no differences in pregnancy outcomes between the pre-pandemic and pandemic periods of COVID-19, suggesting that the virus did not significantly affect pregnancy results [6].

Our study, encompassing a larger participant pool with a higher infection rate, aligned with these findings, indicating that COVID-19 infection in early pregnancy around the FET cycle did not significantly adversely affect early pregnancy outcomes. Notably, the study also suggested that COVID-19 may not cross the placenta readily, reducing the likelihood of vertical transmission in mid- to late pregnancy [2, 17], and there is no evidence that the virus infects embryos in early pregnancy. These findings are consistent with previous research and are in line with current knowledge of the effects of COVID-19 on pregnancy.

A comprehensive analysis of existing literature, including 148 papers collected from PUBMED/MEDLINE and the COVID-19 database of the World Health Organization, indicated that due to the increased time of waiting for fertility treatment and the suspension of fertility services, the pandemic's psychological burden on couples seeking fertility treatment was substantial [12]. The pandemic also had a profound impact on ART live births in the United States, with an estimated loss of approximately 25,143 live births due to the suspension of ART activities and the economic recession associated with the pandemic [18]. Considering these factors, there has been an ongoing debate about the optimal interval between COVID-19 infection and ART treatment to achieve the best pregnancy outcome. The stratified analysis of our study showed that no significant differences were observed in the effects on early embryo implantation and development as well as early pregnancy adverse reactions, between individuals infected with COVID-19 at different periods and uninfected individuals. We believe that there is no need to delay pregnancy plans for possible asymptomatic or mild COVID-19 infections.

Our study, characterized by its large sample size, high population infection rate, and clear infection timeline, stands as a robust contribution to this discussion. By closely examining critical periods related to FET and pregnancy establishment, it provides substantial statistical evidence supporting the notion that asymptomatic or mild COVID-19 infections, even during different periods before and after FET as well as in early pregnancy, do not significantly impact early pregnancy outcomes. This finding holds even in the context of the pandemic when the virus's virulence weakens. Consequently, individuals with asymptomatic or mild infections can confidently pursue pregnancy through natural conception or ART.

Our study has some limitations. The study may be affected by retrospective bias, and there might be limitations in the quality and scope of the available data. In addition, this study has limitations regarding the response of individuals of Asian ethnicity to viral infection. There are no quantitative data on the effect of viral infection, such as measurement of virus titer, assessment of reproductive biological samples (eg follicular fluid, endometrium, etc.). Therefore, more related detailed characteristics should be collected and well-designed studies are needed in the future.

## Conclusion

Our study, carried out during a period when the virulence of the SARS coronavirus had weakened but widespread transmission persisted, suggests that asymptomatic or mild COVID-19 infections occurring around the time of FET do not significantly impact early pregnancy outcomes. Consequently, it may not be necessary for individuals to postpone their pregnancy plans due to the risk of asymptomatic or mild COVID-19 infections.

## Data Availability

The data underlying this article will be shared upon reasonable requests to the corresponding author.

## Abbreviations

ART: Assisted reproductive therapy
BMI: Body mass index
CI: Confidence interval
FET: Frozen embryo transfer
HCG: Human chorionic gonadotropin
IVF: In vitro fertilization
ICSI: Intracytoplasmic sperm injection
TVUS: Transvaginal ultrasound
